# The Value of Safflower Yellow Injection for the Treatment of Acute Cerebral Infarction: A Randomized Controlled Trial

**DOI:** 10.1155/2015/478793

**Published:** 2015-05-17

**Authors:** Le-Jun Li, Yu-Mei Li, Ben-Yu Qiao, Shan Jiang, Xin Li, Hong-Ming Du, Peng-Cheng Han, Jiong Shi

**Affiliations:** ^1^Department of Neurology, Affiliated Lianyungang Hospital of Nanjing University of Chinese Medicine, No. 160 W. Chaoyang Road, Lianyungang 222000, China; ^2^Barrow Neurological Institute, St. Joseph's Hospital and Medical Center, Phoenix, AZ 85013, USA; ^3^Changchun Sanzhen Industry Co., Ltd., Changchun 130000, China

## Abstract

*Background*. Safflower Yellow Injection has been reported as a treatment for acute cerebral infarction in recent studies in China. However, there is a lack of availability of the evidence for the efficacy and safety of Safflower Yellow Injection for the treatment of acute ischemic stroke. So we investigated the effects of Safflower Yellow Injection for the treatment of acute cerebral infarction. *Method*. All subjects were randomly divided into Safflower Yellow Injection group given Safflower Yellow Injection (80 mg) and control group given placebo (0 mg) injection by intravenous drop once daily for 14 days. National Institute of Health Stroke Scale (NIHSS); hemorheological detection; coagulation function; and serum inflammatory markers, tumor necrosis factor-alpha (TNF-*α*), interleukin-1*β* (IL-1*β*), and interleukin-6 (IL-6), were used to investigate the effects before and 14 days after the treatment. *Results*. The scores of NIHSS were decreased on day 7 and day 14 after treatment. The hemorheological index of RBC deformation and RBC aggregation were significantly improved, prothrombin time (PT) increased, and fibrinogen (FIB) and TNF-*α*, IL-1*β*, and IL-6 were decreased in patients treated with Safflower Yellow injection on day 14 after treatment (*P* < 0.05). *Conclusion*. Data suggests that Safflower Yellow Injection therapy may be beneficial for acute cerebral infarction.

## 1. Introduction

Stroke is a leading cause of death and disability worldwide [1]. Despite improvements in acute stroke care-stroke unit care, thrombolysis in appropriately selected patients and early and sustained antiplatelet therapy-many patients only makes a partial or poor recovery after stroke and the major burden of stroke is chronic disability [2]. Therefore, there is a need for treatments that would further improve the efficacy. Clinical research in China, based on traditional Chinese medicine (TCM), has the potential of introducing new treatments for cerebral infarction.

Safflower Yellow Injection was certificated in China by the State Pharmaceutical Administration of China (SPAC) in 2002 (number 2002ZL0176) after being evaluated in clinical trials. The constituent of Safflower Yellow Injection is Safflower Yellow, hydroxysafflor yellow A is a major component of Safflower Yellow [3]. The protective effect of hydroxysafflor yellow A in ischemic stroke has been investigated in previous studies [4], which have shown effects of antithrombosis [5, 6], anticoagulation [7], antioxidation [8], anti-inflammation [9], and anticalcium dysregulation [10].

Safflower Yellow Injection has been reported as a treatment for acute cerebral infarction in recent studies in China [11, 12]. However, there is a lack of good-quality and convincing supporting evidence for the efficacy and safety of Safflower Yellow Injection for the treatment of acute cerebral infarction [4]; no conclusions about efficacy or safety could be drawn [13]. Therefore, our study hypothesis was that patients who underwent a treatment with Safflower Yellow Injection for acute cerebral infarction would have a better therapeutic effect compared to those who did not receive this therapy. So the aim of our study was to assess if giving Safflower Yellow Injection can improve effects of acute cerebral infarction patients in China.

## 2. Materials and Methods 

### 2.1. Study Design

The study was a prospective, single-blinded, and randomized controlled trial. All eligible patients included in the study agree to participate and signed the informed consent form and the study procedures were approved by the Ethical Committee of the Affiliated Lianyungang Hospital of Nanjing University of Chinese Medicine. Full compliance with the Helsinki Declaration was applied during the study. Patients were recruited from the Neurology Department of the Affiliated Lianyungang Hospital of Nanjing University of Chinese Medicine between January 2004 and December 2012.

### 2.2. Inclusion and Exclusion Criteria

All patients meeting the World Health Organization's definition of ischemic stroke were enrolled during the study. Diagnostic criteria of acute cerebral infarction were diagnosed according to diagnostic criteria of various types of cerebrovascular disease [14] and China Guideline for Cerebrovascular Disease Prevention and Treatment. The main inclusion criteria of the trial were diagnosed ischemic stroke patients within 6 h to 14 days of onset; a score of 4–24 points on the National Institute of Health Stroke Scale (NIHSS); adults between 35 and 80 years old; and signed informed consent form. The main exclusion criteria were as follows: after 14 days from onset; a history of previous stroke; hemorrhagic stroke and transient ischemic attacks; other severe diseases such as heart or kidney failure, tumors, and gastrointestinal hemorrhage; and pregnant or lactating women.

### 2.3. Control and Intervention Groups

All patients of both groups were hospitalized for 14 days; we provide baseline treatment for all subjects to keep the respiratory tract unobstructed, preventing and treating aspiration pneumonia, monitoring and treating arrhythmia and ischemic heart disease, normalizing the blood pressure, and controlling blood sugar.

All patients were given conventional antiplatelet aggregation drugs (oral aspirin tablet, 100 mg, once daily) and neuroprotective agent (oral Piracetam tablet 400 mg, tid) therapy, based on conventional treatment; the Safflower Yellow Injection (80 mg) was given to the Safflower Yellow Injection group and placebo (0 mg) injection was given to the control group by intravenous drop once daily for 14 days.

The constituent of Safflower Yellow Injection is Safflower Yellow. The investigational drug Safflower Yellow Injection was supplied as Nankin freeze-dried powder. Each unit contains 80 mg Safflower Yellow. The placebo drug was supplied as blank colorless freeze-dried powder, where each unit contains no Safflower Yellow. Investigational drug and placebo were provided by Changchun Sanzhen Industry Co., Ltd. The Safflower Yellow Injection group intravenously received 80 mg Safflower Yellow dissolved in 250 mL 0.9% sodium chloride or 5% glucose once per day. The control group was given a placebo. For two groups, the course of treatment lasted for 14 days.

### 2.4. Objectives and Outcome Measures

#### 2.4.1. Measurement of NIHSS Score

The NIHSS was measured for stroke severity. The primary outcome measures were the differences in patients' scores on this scale among baseline, day 7, and day 14. The score was assessed by an independent clinical investigator.

#### 2.4.2. Measurement of Hemorheological Detection

Blood samples were collected from ulnar vein before and after the treatment, the index of hemorheolgy included the whole blood viscosity, reduced viscosity, plasma viscosity, hematocrit, and the index of red cell deformity.

Keep 6 mL blood sample in an anticoagulation cuvette mixed with Heparin 100 *μ*L, with LG-R-80 type blood viscosity tester and LG-B-190 type cytomorphosis/aggregation tester, at 37°C ± 0.1°C and detect the whole blood hypsi-tomy coefficient of viscosity (shear rate 150 s^−1^), hypo-tomy coefficient of viscosity (shear rate 5 s^−1^), plasma viscosity, and index of red blood cell deformation and aggregation. The HCT determination used decigram method centrifugation in common temperature with rotary speed 3000 r/min for 30 min.

#### 2.4.3. Measurement of Coagulation Function

Blood samples were collected from ulnar vein before and after the treatment, kept 6 mL blood sample in an anticoagulation cuvette mixed with Heparin 100 *μ*L, centrifuged the sample immediately in common temperature with rotary speed 3000 r/min for 15 minutes and then collected serum and froze it at −80°C to measure. The values of prothrombin time (PT), activated partial thromboplastin time (APTT), fibrinogen (FIB), and international normalized ratio (INR) were detected by using blood coagulation method before and after the treatment.

#### 2.4.4. Measurement of Serum Inflammatory Markers TNF-*α*, IL-1*β*, and IL-6

Peripheral venous blood (10 mL) was collected into sampling tubes without EDTA. Blood was centrifuged at 3500 rpm for 5 minutes to separate the serum. Serum was stored at −80°C until analysis. All assays were performed in a blinded fashion on coded samples. Serum levels of TNF-*α*, IL-1*β*, and IL-6 were measured in duplicate by a sandwich-type enzyme-linked immunosorbent assay (ELISA) technique by using kits from R&D Systems, TNF alpha DTA00C (range of detection: 15.6–1,000 pg/mL), IL-1b DLB50 (range of detection: 3.9–250 pg/mL), and IL-6 D6050.

### 2.5. Statistical Analysis

Statistical analysis was performed using SPSS 12.0 statistics software. Date was presented as mean ± S.D.; baseline variables were analyzed using student's *t*-test. For efficacy variables, they were analyzed using analysis of covariance (ANOVA); comparisons were made between the two groups at baseline, day 7, and day 14. The two-group *t*-test was used separately for each comparison. A level of *P* < 0.05 was considered to be statistically significant.

## 3. Results

### 3.1. Baseline Characteristics

**Figure 1:**
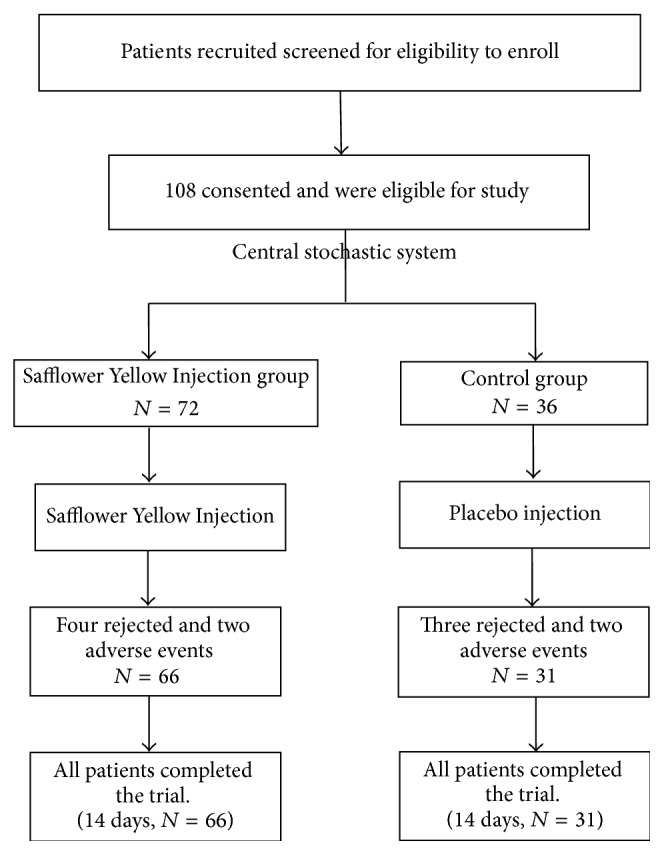
Study design and CONSORT diagram showing the flow of participants.

150 patients were approached and screened for their eligibility to enroll in this study. 108 patients consented and were eligible for the study and signed their informed consent for participation. Patients were randomized by a central stochastic system (2 : 1) to either Safflower Yellow Injection group (*n* = 72) or control group (*n* = 36). Seven patients were rejected during the treatment period for not meeting the inclusion criteria (four from the Safflower Yellow Injection group and three from the control group) and another four patients dropped out for adverse events (two from the Safflower Yellow Injection group and two from the control group). Ninety-seven patients completed the trial, 66 patients in the Safflower Yellow Injection group and 31 patients in the control group (see Figure 1). The characteristics of the Safflower Yellow Injection and control groups are not significantly different at baseline and are given in Table 1.

### 3.2. Efficacy Results

The scores of NIHSS are all differently reduced after treatment for 7 and 14 days. This improvement becomes more apparent with time. After subtracting the baseline effect, the between-group total score is statistically significant (*P* < 0.01) after treatment for 7 and 14 d.

There were no significant differences in the values of hemorheological index between the two groups before the treatment (*P* > 0.05). Safflower Yellow Injection group hemorheological index of RBC deformation and index of RBC aggregation; there were significant differences before and after the treatment (*P* < 0.05). In the control group, only the whole blood viscosity was significantly different before and after the treatment (*P* < 0.05); the remaining hemorheological indices were not significantly different before and after treatment. Safflower Yellow Injection group's whole blood viscosity and plasma viscosity were significantly different after treatment between the two groups (*P* < 0.05). Results are listed in Table 2. The reason may be that Safflower Yellow Injection can improve the values of hemorheological index.

There were no significant differences in the PT, APTT, INR, and FIB values between the two groups before the treatment (*P* > 0.05). The value of PT increased in the Safflower Yellow Injection group after treatment (*P* < 0.05); there was significant difference between the two groups (*P* < 0.05). After the treatment, the values of FIB decreased in the Safflower Yellow Injection group after treatment; there was significant difference between the two groups (*P* < 0.05). Results are listed in Table 3. The reason may be that Safflower Yellow Injection can inhibit both intrinsic and extrinsic coagulations.

There were no significant differences in the serum levels of TNF-*α*, IL-1*β*, and IL-6 between the two groups before the treatment (*P* > 0.05). The serum levels of TNF-*α*, IL-1*β*, and IL-6 in the Safflower Yellow Injection group decreased after treatment (*P* < 0.05); there were significant differences between the two groups (*P* < 0.05). Results are listed in Table 4. The reason may be that Safflower Yellow Injection has anti-inflammatory effects.

### 3.3. Safety and Tolerability

Safflower Yellow Injection for the treatment acute cerebral infarction was well tolerated. Four adverse events were reported during the whole trail (two in the Safflower Yellow Injection group and two from the control group). In the Safflower Yellow Injection group, one patient suffered a transfusion reaction and the other was diagnosed of poststroke depression. Two patients in the control group suffered a recurrence of stroke. All adverse events were considered not to be related to the Safflower Yellow Injection.

## 4. Discussion

Stroke is a sudden onset and acute cerebral vascular disease commonly characterized by focal neurological deficit. It is the most common neurological disease with high incidence, morbidity, mortality, and recurrence. The disease is more common in the elderly, together with myocardial infarction and cancer; these are the three major causes for senile fatality [15].

This clinical study showed that Safflower Yellow Injection is safe and effective for acute cerebral infarction. It effectively improved the stroke symptoms and significantly improved the patient's life competency. The NIHSS is widely used in nearly all large clinical stroke trials to document baseline and outcome severity of neurological impairment [16]. In our study, we found that the scores of NIHSS were significantly decreased in patients treated with Safflower Yellow on day 7 and day 14 after treatment. These results suggest that Safflower Yellow is beneficial in reducing the severity of stroke.

Hemorheological detection is important in the course of disease, including nosogenesis, development, prognosis, and turnover as well as curative effect assessment, especially for prevention of cardiocerebrovascular disease. Brain microcirculation slowdown or stagnation is the main pathological change at the beginning of cerebral ischemia [17]. In our study, we found that the hemorheological index of RBC deformation and RBC aggregation were significantly improved in patients treated with Safflower Yellow Injection on day 14 after treatment. These results suggest that Safflower Yellow Injection has protective effect; the reason may be that Safflower Yellow Injection can reduce whole blood viscosity and plasma viscosity, improve index of RBC deformation, reduce index of RBC aggregation, improve microcirculation, and improve brain following ischemic reperfusion in the acute cerebral infarction; the protective effect of hydroxysafflor yellow A in ischemic stroke has been investigated in previous studies [18].

Coagulation function detection is important in the course of disease, including nosogenesis and curative effect assessment of acute cerebral infarction. In our study, we found that the value of PT increased in the Safflower Yellow Injection group after treatment and the values of FIB decreased in the Safflower Yellow Injection group after treatment, and there were significant differences between the two groups. These results suggest that Safflower Yellow Injection can inhibit both intrinsic and extrinsic coagulations, significantly the prothrombin time and clotting time, and reduce platelet adhesion, thrombosis, and fibrin cross-linking process. Recent studies show that Safflower Yellow inhibits platelet activation factor-induced platelet aggregation, antagonizes platelet activation factor-induced capillary permeability [19, 20], and protects against antithrombin III injuries originated from free radicals [21].

Researchers have demonstrated that the following brain insult cytokine levels are elevated as a result of increased production from inflammatory cells [22] with IL-1, IL-6, and tumor necrosis factor-alpha (TNF-*α*) and being the most studied for stroke [23, 24]. IL-1*β* and TNF-*α* have been associated with exacerbation of injury in stroke while IL-6 has been found to be neuroprotective [25]. In our study, we found that the serum levels of TNF-*α*, IL-1*β*, and IL-6 were significantly decreased in patients treated with Safflower Yellow Injection on day 14 after treatment. These results suggest that Safflower Yellow Injection is beneficial in reducing the serum levels of TNF-*α*, IL-1*β*, and IL-6 after treatment. The reason may be that Safflower Yellow Injection has anti-inflammatory effects; it is considered to be the main source of antioxidant defense in the brain following ischemic reperfusion; the protective effect of hydroxysafflor yellow A in ischemic stroke has been investigated in previous studies [12].

Our data suggest that Safflower Yellow Injection therapy may be beneficial for acute cerebral infarction. In the Chinese studies, Safflower Yellow Injection exhibits a favorable safety profile; there were no serious adverse events recorded and only 2 cases adverse events. This low rate of adverse events may be due to a combination of the fact that the patients were recruited during their recovery phase when their clinical condition had stabilized and due to the method of collection of adverse events in China.

However, there are still some limitations in our study. For example, the size (108 subjects) was not sufficient. The period of observation lasted for only 14 days. To further studies with larger sample size and long-term observations are required. Therefore, we could not rule out the expectation bias because neither the patients nor the therapists were blinded. Some future double-blinded studies would be feasible to be carried out in the future by using Safflower Yellow Injection.

## 5. Conclusions

In summary, our data suggest that Safflower Yellow Injection therapy may be beneficial for acute cerebral infarction. The results presented here invite for further studies with larger sample size, long-term observation, and strict blinding to confirm the efficacy of Safflower Yellow Injection.

## Figures and Tables

**Table 1:** Baseline characteristics of study patients.

Characteristics	Control group		Safflower Yellow Injection group	*P* value
*n* = 97	*n* = 31		*n* = 66
Male	9		28	0.595
Mean age and range, years	58.84 ± 10.18 (30–70)		59.47 ± 10.92 (31–70)	0.564
Body height/cm	Male	170.00 ± 6.69	168.04 ± 6.07	0.573
	Female	159.19 ± 4.82	159.86 ± 4.86	0.554
Body weight/kg	Male	67.56 ± 9.48	67.34 ± 9.85	0.563
	Female	58.73 ± 8.18	60.92 ± 8.44	0.612
Weight index/(kg/m^2^)	23.19 ± 2.49		23.79 ± 2.76	0.963
Disease time/d	5.00 ± 3.55		3.91 ± 2.77	0.925
NIH stroke scale score	7.65 ± 2.32		8.53 ± 3.30	0.556

Values are mean ± S.D.; no significant changes between control group and Safflower Yellow Injection group.

**Table 2:** Comparison of hemorheological index before and after treatment between 2 groups ($\bar{x}\pm s$).

Variables	Control group		Safflower Yellow Injection group	
	*n* = 31		*n* = 66	
	Pretreatment	Posttreatment	Pretreatment	Posttreatment
Whole blood viscosity (mPa·s/150 s^−1^)	5.95 ± 0.80	5.34 ± 0.83^#^	5.92 ± 0.82	5.14 ± 0.85^∗#^
Whole blood viscosity (mPa·s/5 s^−1^)	12.66 ± 2.52	12.57 ± 2.24	12.69 ± 2.64	10.05 ± 2.48^∗#^
Plasma viscosity (mPa·s)	1.82 ± 0.23	1.88 ± 0.26	1.78 ± 0.21	1.42 ± 0.23^∗#^
HCT (%)	49.76 ± 4.80	47.64 ± 4.95	48.53 ± 5.30	47.61 ± 4.77
Index of RBC deformation	0.51 ± 0.01	0.50 ± 0.01	0.51 ± 0.07	0.59 ± 0.03^*^
Index of RBC aggregation	1.21 ± 0.16	1.36 ± 0.19	1.38 ± 0.19	1.21 ± 0.16^*^

Data are expressed as mean ± S.D., ^*^ *P* < 0.05, compared with the pretreatment, ^#^ *P* < 0.05, compared with control group.

**Table 3:** Changes of coagulation and fibrolysis indexes in the patients before and after treatment between 2 groups ($\bar{x}\pm s$).

Variables	Control group		Safflower Yellow Injection group	
	*n* = 31		*n* = 66	
	Pretreatment	Posttreatment	Pretreatment	Posttreatment
PT (s)	10.64 ± 0.58	10.85 ± 0.57	11.02 ± 0.65^#^	13.38 ± 0.71^∗#^
APTT (s)	29.43 ± 4.76	29.21 ± 4.83	29.84 ± 4.81	29.06 ± 5.02
INR	1.04 ± 0.13	0.94 ± 0.12	1.04 ± 0.15	0.94 ± 0.16
FIB (g/L)	3.35 ± 0.61	3.54 ± 0.70	3.57 ± 0.73	2.07 ± 0.19^∗#^

Data are expressed as mean ± S.D., ^*^ *P* < 0.05, compared with the pretreatment, ^#^ *P* < 0.05, compared with control group.

**Table 4:** Comparison of serum levels of TNF-*α*, IL-1*β*, and IL-6 before and after treatment between 2 groups ($\bar{x}\pm s$).

Variables	Control group		Safflower Yellow Injection group	
	*n* = 31		*n* = 66	
	Pretreatment	Posttreatment	Pretreatment	Posttreatment
TNF-*α*	39.45 ± 5.62	40.24 ± 1.55	39.82 ± 4.71	30.85 ± 3.10^∗#^
IL-1*β*	44.16 ± 4.32	40.07 ± 1.44	44.69 ± 4.24	28.85 ± 1.68^∗#^
IL-6	669.92 ± 61.93	711.28 ± 31.86	689.88 ± 48.92	558.82 ± 38.03^∗#^

Data are expressed as mean ± S.D., ^*^ *P* < 0.05, compared with the pretreatment, ^#^ *P* < 0.05, compared with control group.
