# Corrigendum to “The Value of Safflower Yellow Injection for the Treatment of Acute Cerebral Infarction: A Randomized Controlled Trial”

**DOI:** 10.1155/2016/4270317

**Published:** 2016-09-18

**Authors:** Le-Jun Li, Yu-Mei Li, Ben-Yu Qiao, Shan Jiang, Xin Li, Hong-Ming Du, Peng-Cheng Han

**Affiliations:** ^1^Department of Neurology, Affiliated Lianyungang Hospital of Nanjing University of Chinese Medicine, No. 160 W. Chaoyang Road, Lianyungang 222000, China; ^2^Barrow Neurological Institute, St. Joseph's Hospital and Medical Center, Phoenix, AZ 85013, USA; ^3^Changchun Sanzhen Industry Co., Ltd., Changchun 130000, China

In the article titled “The Value of Safflower Yellow Injection for the Treatment of Acute Cerebral Infarction: A Randomized Controlled Trial” [1], Dr. Jiong Shi was incorrectly included as an author. The corrected author list is shown above.

## References

[1] Li L.-J., Li Y.-M., Qiao B.-Y. (2015). The value of Safflower Yellow Injection for the treatment of acute cerebral infarction: a randomized controlled trial. *Evidence-Based Complementary and Alternative Medicine*.

